# Inhibition of HCV 3a genotype entry through Host CD81 and HCV E2 antibodies

**DOI:** 10.1186/1479-5876-9-194

**Published:** 2011-11-10

**Authors:** Usman A Ashfaq, Muhammad Qasim, Muhammad Z Yousaf, Muhammad Tariq Awan, Shah Jahan

**Affiliations:** 1 Division of Molecular Medicine, National Centre of Excellence in Molecular Biology, University of the Punjab, Lahore, Pakistan; 2 Institute of Biochemistry and Biotechnology, University of Veterinary and Animal Sciences, Lahore, Pakistan; 3 Applied and Functional Genomics Lab, Centre of Excellence in Molecular Biology, University of the Punjab, Lahore, Pakistan

## Abstract

**Background:**

HCV causes acute and chronic hepatitis which can eventually lead to permanent liver damage hepatocellular carcinoma and death. HCV glycoproteins play an important role in HCV entry by binding with CD81 receptors. Hence inhibition of virus at entry step is an important target to identify antiviral drugs against HCV.

**Methods and result:**

The present study elaborated the role of CD81 and HCV glycoprotein E2 in HCV entry using retroviral pseudo-particles of 3a local genotype. Our results demonstrated that HCV specific antibody E2 and host antibody CD81 showed dose- dependent inhibition of HCV entry. HCV E2 antibody showed 50% reduction at a concentration of 1.5 ± 1 μg while CD81 exhibited 50% reduction at a concentration of 0.8 ± 1 μg. In addition, data obtained with HCVpp were also confirmed with the infection of whole virus of HCV genotype 3a in liver cells.

**Conclusion:**

Our data suggest that HCV specific E2 and host CD81 antibodies reduce HCVpp entry and full length viral particle and combination of host and HCV specific antibodies showed synergistic effect in reducing the viral titer.

## Background

HCV is a major health problem that infects 350 million people worldwide and 10 million people in Pakistan [1]. HCV infection is mainly restricted to hepatocytes, and since most of the infected individuals fail to spontaneously clear the virus from the liver, this leads to a chronic infection that can evolve towards liver fibrosis, cirrhosis and hepatocellular carcinoma over a period of decades [2]. The current standard therapy is Pegylated interferon and ribavirin, which shows poor tolerability and is only capable of attaining a sustained viral response in half of patients due to resistance mutations, adverse side effects and high cost [3].

HCV is a small enveloped virus with a positive-sense, single-stranded RNA genome that encodes a large polyprotein of 3010 amino acids. The polyprotein is co- and post-translationally processed by cellular and virally encoded proteases to produce four structural (Core, E1, E2 and P7) and six non-structural proteins (NS2, NS3, NS4A, NS4B, NS5A, NS5B) [4,5]. Among the structural protein, HCV envelop protein E1 and E2 are highly glycosylated and play an important role in cell entry. HCV NS3 serine protease and NS5b RNA dependent RNA polymerase play an important role in replication. HCV NS3 serine protease, NS5B RNA-dependent RNA polymerase and HCV structural proteins are vital targets for antiviral drug development.

Due to the absence of suitable animal model and competent in-vitro cell culture system the mechanism of HCV cell entry was unrevealed after a long time. Recently, different groups have studied HCV replication in serum infected liver cell lines which mimics the naturally occurring HCV virions biology and kinetics of HCV infection in humans hepatocytes [6-9]. HCV envelop glycoproteins E1 and E2 are involved in HCV entry, fusion and defense against neutralization by envelop-specific host antibodies [10-13]. E2 glycoprotein works as a key component in interaction between the virus and its major cellular receptors i.e., CD81, SR-BI and CLDN1 [13].

CD81 is a 26-kDa surface protein composed of four hydrophobic transmembrane domains and two hydrophilic extracellular domains (EC1 and EC2) [14]. Like other members of the tetraspanin superfamily, CD81 is expressed in a range of organisms, including mouse and chimpanzee, and on most human tissues apart from red blood cells and platelets [15]. The cytoplasmic and transmembrane domains as well as small extracellular loop of CD81 are highly conserved between species, while the large extracellular domain varies considerably both in length and sequence, thus contributing to species-specific interactions. Cross-linking experiments have shown that human CD81 mediates a number of signal transduction events involved in the regulation cell proliferation, morphology, differentiation, adhesion, and motility [14]. Human CD81 was identified to interact with soluble HCV E2 and virus in serum and was proposed to play a role in HCV entry [16,17]. HCV E2 envelop protein interact with CD81, scavenger receptor type B class 1 protein (SRB-1) and high density lipoprotein (HDL) binding molecule [17,18]. CD81 monoclonal antibody can inhibit entry of HCVpp to cells [19].

The present study was designed to explore the anti-HCV effect of Host CD81 and HCV specific E2 antibodies. For this purpose, HCVpp of 3a local genotype were produced by transfecting three vectors in HEK 293 T cells and were used to infect liver cells in the presence and absence of host and HCV specific antibodies.

## Materials and methods

### Serum Sample Collection

HCV-3a patient's serum sample used in this investigation was obtained from the CAMB (Center for Applied Molecular Biology) diagnostic laboratory, Lahore, Pakistan. Serum sample was stored at -80°C prior to viral inoculation experiments. Quantification and genotype was assessed by CAMB diagnostic laboratory, Lahore, Pakistan. Patient's written consent and approval for this study was obtained from Institutional Ethics Committee.

### Cell lines

Huh-7 and HEK 293 T cells were cultured in Dulbecco's Modified Eagle medium (DMEM) supplemented with 10% fetal calf serum, 100 IU/ml penicillin and 100 μg/ml streptomycin, at 37°C in an atmosphere of 5% CO_2_. Huh-7 cells were kindly provided by Dr. Zafar Nawaz (Biochemistry and Molecular Biology Department, University of Miami, USA). CHO was provided by Dr. Ahmad Usman Zafar (Biopharmaceutical Lab, CEMB, Pakistan).

### Plasmids

The pcDNA-E1E2 expression vector encoding the E1 and E2 glycoproteins (171-746) of HCV genotype 3a, was generated by inserting into a nonpackageable, CMV promoter-driven expression construct (provided by Shazia Rafique, virology lab, CEMB, Pakistan). The CMV-Gag-Pol murine leukemia virus (MLV) packaging construct, encoding the MLV *gag *and *pol *genes, and the pTG-Luciferase plasmid provided by Dr. Jaean Dubison, France.

### Production of HCVpp and infection

HCVpp were produced by co-transfection of 293-T cells with equal amounts of three expression vectors as described previously [10]. Supernatants containing pseudo-particles were harvested 48 h later, filtered through 0.45 μm pore-sized membranes and stored at -80°C before use in infection of Huh7 cells in the presence and absence of CD81 and E2 antibodies.

### Inhibition of HCV through host CD81 and HCV specific E2 antibodies

Huh-7 cells were maintained in 6-well culture plates to semi-confluence, washed twice with serum-free medium and then HCV E2 antibody and CD81 antibody incubated with liver cells at 37°C for 1 h. After 1 h cells were infected with 2 × 10^5 ^copies of HCV 3a genotype per well and incubated for additional 24 h. After 24 h, cells and total RNA was isolated by using Gentra RNA isolation kit (Gentra System Pennsylvania, USA) according to the manufacturer's instructions. Briefely, cells were lysed with cell lysis solution containing 5 μl internal control (Sacace Biotechnologies Caserta, Italy). RNA pallet was solubilized in 1% DEPC (Diethyl pyrocarbonate treated water). HCV RNA quantifications were determined by Real Time PCR Smart Cycler II system (Cepheid Sunnyvale, USA) using the Sacace HCV quantitative analysis kit (Sacace Biotechnologies Caserta, Italy) according to the manufacturer's instructions.

#### Formula for the calculation of HCV RNA concentration

Following formula was used to calculate the concentration HCV RNA of each sample.

$$\frac{\text{Cy}3\text{STD}∕\text{Res}}{\text{Fam}\text{.}\;\text{STD}∕\text{Res}}\times \text{coefficient}\;\text{IC = IU}\;\text{HCV/mL}$$

IC = internal control, which is specific for each lot.

### Statistical Analysis

All statistical analysis was done using SPSS software (version 16.0, SPSS Inc). Data are presented as mean ± SD. Numerical data were analyzed using student's t-test and ANOVA. P value < 0.05 was considered statistically

## Results

### Anti infectivity effect of CD81 and HCV E2 antibody against HCVpp and HCV infected serum

**Figure 1:**
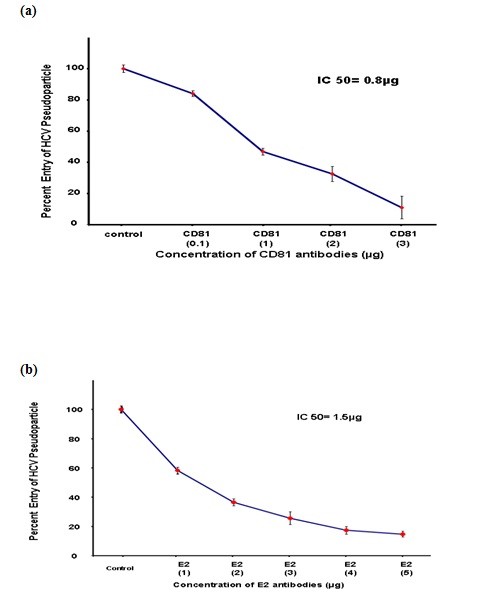
**Dose dependent inhibition of HCVpp of 3a genotype**. HCVpp was produced in HEK 293 T cells and collected in media after filtration in 0.45 micron filter.(a) CD81 monoclonal antibody were incubated with Huh-7 cells at 37°C for 1 h. (b) HCV E2 monoclonal antibody incubated with HCV Pseudo particles at 37°C for 1 hr. After 1 hrs Huh-7 cells were infected with pseudo particle of HCV 3a genotype in the presence and absence of different concentrations of CD81 and HCV E2 antibody and incubated for 3 hrs. After 24 hrs cells were lysed and luciferase activity was determined by using a luminometer. Luciferase activity is not reported as an absolute value, but is calculated relative to the 'no drug' condition and reported on the *y*-axis as a percentage. Results are represented as the average and standard error for three independent experiments. P value > 0.05 vs control was considered as statistically significant.

**Figure 2:**
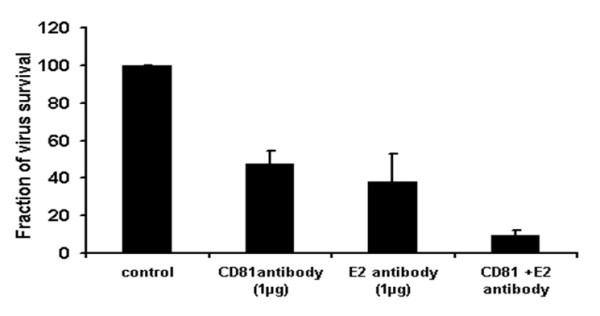
**Antiviral effect of CD81 and HCV E2 antibodies against HCV 3a genotype in liver cells**. HCV E2 antibody was incubated with 2 × 10^5 ^HCV 3a virus and CD81 antibody incubated with liver cells at 37°C for I h. After 1 h cells were infected with 2 × 10^5 ^copies of HCV 3a genotype per well and incubated for additional 24 h. At the end of incubation period, total RNA was extracted by Gentra kit, and the levels of HCV RNA remaining were determined, by real time Quantitative RT-PCR assay and are shown as percentage of HCV RNA survival in cells. Results are represented as the average and standard error for three independent experiments. P value > 0.05 vs control was considered as statistically significant.

In this study, we targeted HCV E2 protein with HCV E2 specific antibody and CD81 receptor with CD81 specific antibody to check the role of HCV E2 and CD81 receptor in HCV entry. HCVpp of genotype 3a were incubated with different concentration of HCV E2 monoclonal antibody at 37°C for 1 h and Huh-7 cells were incubated with different concentration of CD81 antibody at 37°C for 1 h. After 1 h Huh-7 cells were infected with HCVpp. After 24 h cells were lysed and luciferase activity was determined by using a luminometer. CD81 antibody exhibited dose-dependent reduction of HCVpp entry and resulted in 50% reduction in HCVpp entry at 0.8 ± 1 μg concentration and 90% reduction in HCVpp entry at 3 ± 1 μg concentration as shown in Figure 1a. HCV E2 antibody also showed dose-dependent reduction of HCVpp entry and exhibited 50% reduction in HCVpp entry at 1.5 ± 1 μg concentration as shown in Figure 1b. CD81 and HCV E2 antibody also showed reduction of HCV serum infectivity in liver cells. CD81 and HCV E2 antibody resulted in 53% and 63% reduction of HCV infected serum respectively. Moreover, combination of CD81 and HCV E2 antibody synergistically exhibited 91% reduction of HCV serum infectivity as shown in Figure 2.

## Discussion

HCV entry represents an attractive target for drug discovery from a mechanistic point of view, with opportunities to prevent multiple virus-receptor interactions and to interfere with virus-cell membrane fusion [20]. Each of these steps, although not completely defined, is likely mediated by the HCV E1 and/or E2 envelope glycoproteins. *In vitro*, proof-of-concept for inhibiting the HCV entry process has been demonstrated using Glanthus nivalis Agglutinin (GNA) that targets the *N*-linked glycans of the viral envelope proteins and prevents E2-CD81 interaction [21], neutralizing antibodies directed against the HCV E1 and E2 proteins [22-27], antibodies against cellular receptors CD81 [9,11,28-31], SR-BI and agents that block endosomal acidification [32]. In this study, HCVpp of local HCV genotype 3a were produced to study early entry steps mediated by HCV envelope glycoproteins. This assay is based on the quantification of retroviral DNA synthesis, which occurs soon after the fusion of the retroviral particle with a cellular membrane. Presumably, this assay is only dependent on the entry steps mediated by the heterodimer E1E2 (binding, endocytosis, and fusion) and on the activity of the reverse transcriptase of the HCVpp retroviral core. Furthermore, data obtained with HCVpp was also confirmed with the infection of whole virus of HCV genotype 3a in liver cells.

Previous evidence suggests that cell-mediated immunity play an important role in clearance and control of HCV replication in acute infection [33]. It is not necessary that all antibodies of HCV E2 envelop protein inhibit virus entry by neutralization of viral infection. However, majority of E2 antibodies that demonstrated broad neutralization of infection are directed against conformational epitopes within E2 envelop protein [26,27]. Our data showed that antibodies against HCV E2 envelop protein inhibit HCV entry of 3a local genotype in a dose-dependent manner and resulted in 50% reduction in HCVpp entry at a concentration of 1.5 ± 1 μg.

HCV entry is a multiple-step process and several host proteins have been identified as receptors, including CD81, SRBI, Claudin I and occludin [31,34,35]. In this study we showed that CD81 is required for HCV glycoprotein dependent entry of HCVpp. Our data exhibited that HCVpp entry is blocked in hepatoma cell line with CD81 specific monoclonal antibody. Several reports also illustrated that CD81 is required for HCVpp entry. Silencing CD81 with CD81 specific siRNA has blocked entry of HCVpp [28].

HCV infects liver cells, replicates efficiently and continuously in liver derived Huh-7 cells [36]. Huh-7 cells are most widely used for liver associated diseases and fundamental studies for the development of antiviral agents against HCV as infectious cell culture system [10,37-39]. Recently different groups have studied the HCV replication in serum infected liver cell lines which mimics the naturally occurring HCV virions biology and kinetics of HCV infection in human. We infected Huh-7 cells with native viral particles from HCV-3a positive serum using the same protocol as describe by El-Awady et al., and Zekri et al [7,40]. Our data proved that CD81 and HCV E2 antibody exhibited 53% and 63% reduction of HCV infected serum respectively. In addition, combination of CD81 and HCV E2 antibody synergistically showed 91% reduction of HCV serum infectivity.

## Conclusion

In conclusion, our results demonstrated that HCV E2 and host CD81 dose dependently inhibit HCVpp and full length viral particle entry in liver cells. Moreover, combination of E2 and CD81 antibodies showed synergistic effect in preventing HCV infection.

## Abbreviations

**HCV**: Hepatitis C virus; **Huh-7**: Human Hepatoma Cell line; **HCVpp**: HCV pseudoparticles

## Competing interests

The authors declare that they have no competing interests.

## Authors' contributions

UAA performed lab work and manuscript write up. MQ, MZY, MTA and SJ helped me in writing the manuscript. All the authors read and approved the final manuscript.

## Authors' information

Usman A Ashfaq (PhD Molecular Biology), Muhammad Qasim (PhD Molecular Biology), Muhammad Z Yousaf (PhD Molecular Biology), Muhammad Tariq Awan (PhD Molecular Biology), Shah Jahan (PhD Molecular Biology)
